# Phylogenomic analysis of vertebrate thrombospondins reveals fish-specific paralogues, ancestral gene relationships and a tetrapod innovation

**DOI:** 10.1186/1471-2148-6-33

**Published:** 2006-04-18

**Authors:** Patrick McKenzie, Seetharam C Chadalavada, Justin Bohrer, Josephine C Adams

**Affiliations:** 1 Cleveland Clinic Lerner College of Medicine of Case Western Reserve University, Cleveland Clinic Foundation, Cleveland, OH 44195, USA; 2 Dept. of Cell Biology, Lerner Research Institute, Cleveland Clinic Foundation, Cleveland, OH 44195, USA

## Abstract

**Background:**

Thrombospondins (TSPs) are evolutionarily-conserved, extracellular, calcium-binding glycoproteins with important roles in cell-extracellular matrix interactions, angiogenesis, synaptogenesis and connective tissue organisation. Five TSPs, designated TSP-1 through TSP-5, are encoded in the human genome. All but one have known roles in acquired or inherited human diseases. To further understand the roles of TSPs in human physiology and pathology, it would be advantageous to extend the repertoire of relevant vertebrate models. In general the zebrafish is proving an excellent model organism for vertebrate biology, therefore we set out to evaluate the status of TSPs in zebrafish and two species of pufferfish.

**Results:**

We identified by bioinformatics that three fish species encode larger numbers of TSPs than vertebrates, yet all these sequences group as homologues of TSP-1 to -4. By phylogenomic analysis of neighboring genes, we uncovered that, in fish, a TSP-4-like sequence is encoded from the gene corresponding to the tetrapod TSP-5 gene. Thus, all TSP genes show conservation of synteny between fish and tetrapods. In the human genome, the TSP-1, TSP-3, TSP-4 and TSP-5 genes lie within paralogous regions that provide insight into the ancestral genomic context of vertebrate TSPs.

**Conclusion:**

A new model for TSP evolution in vertebrates is presented. The TSP-5 protein sequence has evolved rapidly from a TSP-4-like sequence as an innovation in the tetrapod lineage. TSP biology in fish is complicated by the presence of additional lineage- and species-specific TSP paralogues. These novel results give deeper insight into the evolution of TSPs in vertebrates and open new directions for understanding the physiological and pathological roles of TSP-4 and TSP-5 in humans.

## Background

The thrombospondins (TSPs) are extracellular, calcium-binding glycoproteins with roles in cell-extracellular matrix interactions, angiogenesis and tumor growth, synaptogenesis, and the organization of connective extracellular matrix (ECM) [1-4]. TSPs have been well-conserved in animal evolution as ECM components. The *Drosophila melanogaster *genome encodes a single TSP which is dynamically expressed during embryogenesis at sites of tissue remodeling including imaginal discs, precursor myoblasts, and muscle/tendon attachment sites [5]. A TSP of the kuruma prawn, *Marsupenaeus japonicus*, is a major component of oocyte cortical rods, specialized storage structures for ECM components that are released to cover the egg upon fertilization [6]. Five TSPs, designated TSP-1 to TSP-5, are encoded in the human and mouse genomes, all of which have dynamic and specific patterns of expression during embryogenesis and in adult life (reviewed in [3]). Mouse gene knockouts prepared for TSP-1, TSP-2, TSP-3, and TSP-5 have demonstrated distinct roles for these family members in normal tissue development and/or adult physiology and pathology [7-10].

All TSPs have the same domain architecture in their C-terminal regions, consisting of EGF domains, a series of calcium-binding, TSP type 3 repeats and a globular C-terminus that is related in structure to L-type lectins [11,12]. The entire C-terminal region forms a structural unit in which calcium-binding has a critical role in the physical conformation and functional properties [13-15]. Many TSPs also contain a globular amino-terminal domain that folds as a laminin G-like domain [16]. Vertebrate TSPs can be grouped into two structural subgroups, A and B, according to their molecular architecture and oligomerization status [17]. TSP-1 and TSP-2, in subgroup A, are distinguished by the presence of a von Willebrand factor type_C (vWF_C) domain and three thrombospondin type 1 repeats adjacent to their N-terminal domains and oligomerize as trimers. TSP-3, TSP-4 and TSP-5, (TSP-5 is also known as cartilage oligomeric matrix protein, COMP [18]), in subgroup B lack these domains, contain an additional EGF domain and assemble as pentamers [19-21]. TSP-5/COMP also lacks a distinct N-terminal domain. The multidomain and multimeric organization of TSPs mediate their complex and tissue-specific physiological functions that are known in mammals.

Importantly, TSP family members have multiple roles in inherited and acquired human disease. TSP-5/COMP is most highly expressed in cartilage and point mutations in its type 3 repeats and L-lectin domain are causal in pseudoanchrondroplastic dysplasia (PSACH) and some forms of multiple epiphyseal dysplasia (MED) (OMIM 117170 and 132400). These mutations cause functional perturbation through effects on calcium-binding and intra- or intermolecular interactions that impair both the post-translational processing and secretion of TSP-5/COMP and its interactions with other ECM molecules in cartilage ECM (reviewed in [22]). Single nucleotide polymorphisms (SNPs) in the coding sequences of TSP-1 and TSP-4 are associated with increased risk of familial premature heart disease [23,24]. These coding SNPs also alter the calcium-binding and physical properties of TSP C-terminal regions, correlating with altered interactions with and signaling effects on vascular cells [25-27]. In contrast, a SNP in the 3' untranslated region of TSP-2 has protective effects against myocardial infarction [23]. Also indicative of a protective role in the myocardium, TSP-2 gene knockout mice have increased susceptibility to angiotensin II-induced cardiac failure [28]. TSP-1 and TSP-2 are also known as natural inhibitors of angiogenesis that can suppress the vascularization of tumors by triggering microvascular endothelial cell apoptosis by binding CD36 (reviewed in [2]). Down-regulation of TSP-1 has been documented in certain human tumors and the expression level of TSP-1 impacts on tumor growth [29-31]. A TSP-1 peptide mimetic is in clinical trial as a novel anti-cancer therapy [32].

To date, the functions of TSPs *in vivo *have been examined experimentally only in mice, yet in general the zebrafish is proving an excellent model for analysis of the musculoskeletal and cardiovascular systems and has the definite advantages of a faster lifecycle, large numbers of progeny, and accessibility of all embryonic stages for experimental analysis and imaging [33,34]. However, despite an intense research focus on mammalian TSPs, the phylogeny of TSPs in other vertebrates is not well understood. With these considerations in mind, we have combined molecular phylogenetic and phylogenomic approaches to address whether fish would be appropriate model organisms for future experimental study of TSPs in relation to their roles in human disease.

## Results

### An overview of TSPs in vertebrate subphyla

Five separate TSP-encoding genes have been identified in human and mouse. To prepare a full TSP dataset that included other vertebrate subphyla, we searched the sequenced genomes of the chicken *Gallus gallus *[35]; the fish *Takifugu rubripes *(marine pufferfish) [36]; *Tetraodon nigroviridis *(freshwater pufferfish) [37]; *Danio rerio *(zebrafish) [38,39] and the amphibian *Xenopus tropicalis *([40]; genome assembly v4.1 at JGI), with either human TSP-1 or TSP-5 as the query sequence. TBLASTN searches were made against the genomic sequences, and BLASTP searches were carried out against databases of genome-predicted proteins, if available. These approaches identified that the *G. gallus *and *X. tropicalis *genomes each encode five TSPs (Table 1). These were identified as orthologues of TSPs 1–5 of human and mouse by BLASTP search against the non-redundant protein database at NCBI. The lack of an amino-terminal globular domain is a distinctive feature of mammalian TSP-5/COMP, and we confirmed that this domain was indeed absent from *G. gallus *and *X. tropicalis *TSP-5 [see Additional File 1]. Each of the identified *G. gallus *and *X. tropicalis *TSPs also corresponded to a transcribed sequence, as established by identification of exactly-matching cDNAs, either from published sequences or from expressed sequence tags (ESTs) in the NCBI dbEST database (data not shown). Four of the chicken TSP genes have been mapped and, as in human and mouse, each is located on a different chromosome [41] (Table 1). The *X. tropicalis *genome is currently assembled in scaffold form only.

**Table 1:** Dataset of vertebrate TSPs compiled from fully-sequenced genomes and genome-predicted proteins for this study.

TSP	TSP-1	TSP-2	TSP_3	TSP-4	TSP-5/COMP
Species					
T. rubripes	a: SINFRUP00000078556 (scaffold_376) b: SINFRUP00000091179 (scaffold_260)	SINFRUP00000052212 (scaffold_1131)	SINFRUP00000068259 (scaffold_3664)	a:SINFRUP00000074734 (scaffold_1187) b:SINFRUP00000057986 (scaffold_305)	
T.nigroviridis	a :CAG03524 (chro 14) b :CAG10667 (Chro 10)	CAG09456 (chro 17)		a:CAG07859 (chro 12) b:CAG00605 (chro 1) c:CAG06350 (chro 4)	
D.rerio	CAI20599 (chro 20)	a:ENSDARP00000057230 (chro 13) b:ENSDARP00000030477 (chro 12)	a:NP_775332 (chro 16) b:XP_699985 (chro19)	a:NP_775333 (unmap*) b:XP_690679 (unmap, Zv5 NA2846) c:ENSDARP00000022442 (partial sequence, chro 5)	
X.tropicalis	Xt4l278562lestExt_gw1.C_2730021 (scaffold_273)	Xt4l302353le_gw1.2.579.1 (scaffold_2)	NP_001011401 (scaffolds_3931 and _3512)	Xt4l458958lestExt_fgenesh1_pg.C_5790005 (scaffold_579)	Xt4l387829le_gw1.580.20.1 (scaffold_580)
G.gallus	XP_421205 (chro 5)	XP_419599 (chro 3)	L81165 (unmap)	XP_424763 (chro Z)	XP_418238 (chro 28)
M.musculus	A40558 (chro2 band F)	Q03350 (chro17 band F)	NP_038719 (chro 3 band E3)	NP_035712 (chro13-52)	AF033530 (chro8-22)
H.sapiens	P07996 (chro15q15)	P35442 (chro6q27)	P49746 (chro1q21)	P35443 (chro5q23)	P49747 (chro19p13.1)

Predicted proteins are assigned by their BLASTP bit scores against the five human TSPs. Proteins are identified according to GenBank accession numbers where available. In the case of *T. rubripes*, the *Fugu *genome-predicted protein identifiers are given; for *D. rerio*, sequences not in GenBank are identified by their Ensembl DARP numbers. *X. tropicalis *proteins are identified according to the genome assembly 4.1 Gene Models (proteins) identifiers. Chromosomal locations are given in brackets for the physically mapped genomes and genomic scaffold numbers are provided for *T. rubripes *(as per *Fugu *Genome sequencing consortium) and *X. tropicalis *(as per JGI genome assembly v4.1). *, mapping uncertain until a finished clone for the region is completed in zebrafish genome assemby 5 (Wellcome Trust Sanger Institute).

In contrast, our searches of the three fish genomes identified 6 to 8 TSPs encoded in each genome (Table 1). The *T. rubripes *and *T. nigroviridis *genomes each encoded six TSP sequences. By BLASTP searches, these sequences grouped as homologues of TSP-1, TSP-2, TSP-3 or TSP-4 (Table 1). In the case of *T. rubripes*, two sequences were most similar to TSP-1, two sequences were most similar to TSP-4, and the remaining two sequences were most similar to TSP-2 or TSP-3, respectively. Each TSP-encoding sequence was located on a different genomic scaffold (Table 1). In *T. nigroviridis*, two sequences were most closely-related to TSP-1, one to TSP-2, and three were most similar to TSP-4. The *T. nigroviridis *genome has been mapped physically [37] and each of the six TSPs were located on a different chromosome (Table 1).

From the zebrafish genome, assembly Zv5 of August 2005, we identified 8 TSP-like sequences. As in the pufferfish, the *D.rerio *TSP sequences appeared homologous to either TSP-1, -2, -3 or -4 (Table 1). Two of the TSPs corresponded exactly to published sequences for *D. rerio *TSP-3 and TSP-4 predicted from cDNA [42] (Table 1). The other six sequences encoded a predicted TSP-1, two TSP-2s, another TSP-3, and two other predicted TSP-4-like polypeptides. The six mapped genes are encoded at separate loci (Table 1). We took advantage of the large number of ESTs available from zebrafish in dbEST (634, 605 as of August 1, 2005) to establish whether all eight TSPs are transcribed : ESTs of 100 % identity were identified for six of the TSPs, but not for the TSP-2 on chromosome 12 or the partial TSP-4c sequence on chromosome 5. Our further analysis therefore focused on the six TSPs that are definitely transcribed.

### Relationship of fish and tetrapod TSPs : assessment by molecular phylogeny

**Figure 1:**
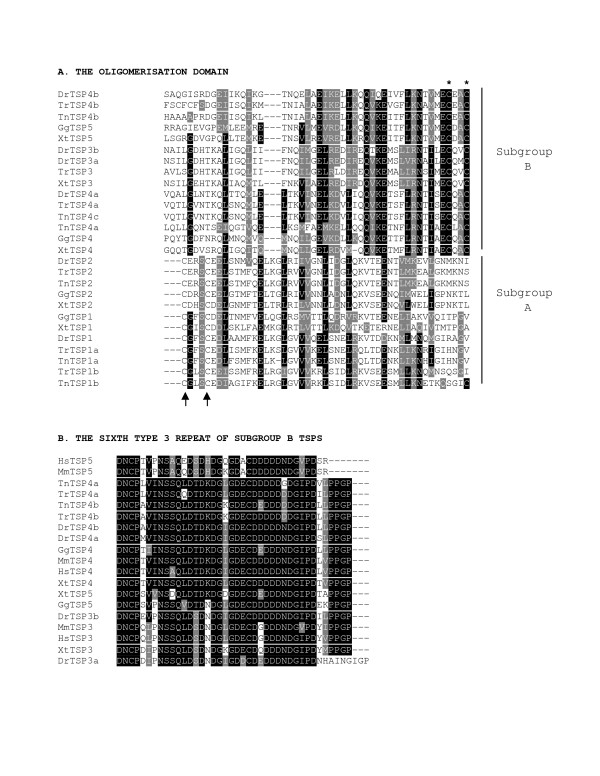
**A, The oligomerization domains of fish and representative tetrapod TSPs**. Oligomerization regions identified by the COILS program were aligned by the program TCOFFEE. The positions of the two adjacent conserved cysteine residues are indicated with arrows (subgroup A) or asterisk (subgroup B). B, Alignment of the sixth type 3 repeat of vertebrate subgroup B TSPs, displaying the PPGP motif that is absent from mammalian TSP-5s. Each alignment is presented in Boxshade 3.2. Black shading indicates identical amino acids, grey shading indicates conservative substitutions and white background indicates unrelated amino acids. Key : Dr = *Danio rerio*; Gg = *Gallus gallus*; Hs = *Homo sapiens*; Mm = *Mus musculus*; Tn = *Tetraodon nigriviridis*; Tr = *Takifugu rubripes*; Xt = *Xenopus tropicalis*.

In view of the larger numbers of TSPs in each fish genome and the many TSP-4-like sequences, we assessed the relationships of the predicted proteins to tetrapod TSP-1 to -5 in more detail. A signature of TSP subgroups A and B is associated with the heptad-repeat coiled-coil domain that mediates oligomerization of TSP subunits. Subgroup A and B family members differ in the placement of two cysteine residues that assist oligomerization by forming inter-subunit disulfide bonds : these cysteines are located before the coiled-coil domain in subgroup A TSPs and after the coiled-coil in subgroup B TSPs [5,19-21]. We aligned the available predicted heptad-repeat regions (identified by the COILS program) of the TSPs from fish, *X. tropicalis *and *G. gallus *and examined the positioning of any adjacent paired cysteine residues. All the fish TSP sequences contained adjacent paired cysteine residues that aligned in the expected A or B patterns with those of the frog and chicken TSPs (Fig. 1A). Thus, with regard to oligomerization, fish TSPs are identical to tetrapod TSPs.

We examined the domain architecture of the fish TSPs through the CDD, SMART and InterPro databases. All the fish-encoded sequences identified as homologous to mammalian TSP-1 and TSP-2 contained vWF_C and TSP type 1 domains and were thus confirmed as belonging to TSP subgroup A. Those identified as subgroup B homologues on the basis of the oligomerization domain lacked these domains and included an additional EGF domain. All known TSPs contain at least one EGF domain with a consensus sequence for beta-hydroxylation of an asparagine residue, indicative of a capacity for calcium-binding, [43], and this trait was conserved in all the newly-identified fish TSPs (data not shown). Human and mouse TSP-3 and TSP-4 are distinguished from TSP-5 by the presence of a 4-amino acid insert motif, PPGP, at the end of the sixth type 3 repeat that may alter calcium-binding activity [44]. Examination of the sixth type 3 repeat of the subgroup B TSPs in our dataset revealed that PPGP motifs were present in the fish TSP-3, TSP-4a and TSP-4b sequences and also, unexpectedly, in each of TSP-3, TSP-4 and TSP-5 from *X. tropicalis *and chicken. *D. rerio *TSP-3a has an unusually long repeat that contains a variant motif, GIGP (Fig. 1B). These results reveal that the absence of the PPGP motif from mammalian TSP-5 is a secondary trait that is not inherent to all forms of TSP-5.

**Figure 2:**
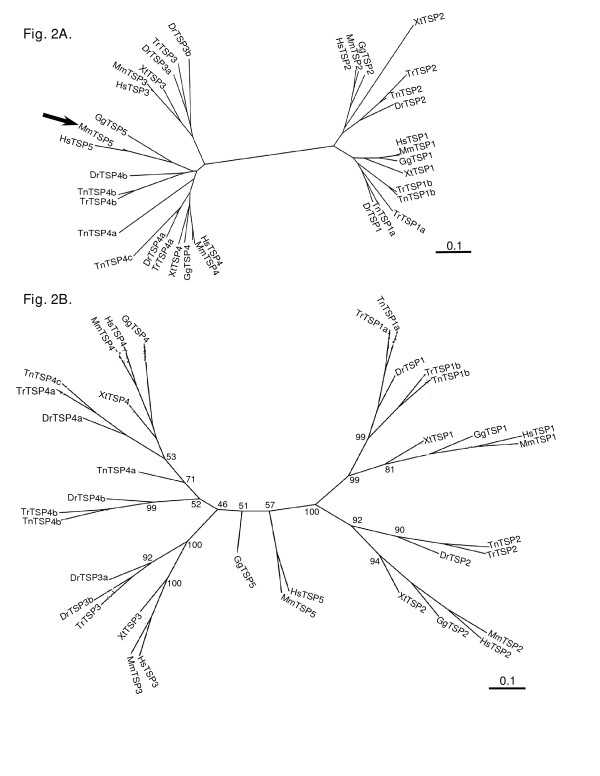
**Protein sequence relationships of the C-terminal regions of fish and tetrapod TSPs**. A, Sequences corresponding to aa 674–1152 of human TSP-1 were aligned in CLUSTALW. The unrooted tree was prepared in Phylip DRAWTREE and is presented in Phylodendron. The cluster of tetrapod TSP-5 sequences within the broad TSP-4/TSP-5 grouping is arrowed. B, The same sequences were aligned in TCOFFEE and analyzed by the maximum-likelihood method, PHYML, including 100 bootstrap cycles. Bootstrap values are given for the major internal nodes: values above 70 are taken to indicate stability of the branchpoint. Scale bars = 0.1 substitutions/site. Key : Dr = *Danio rerio*; Gg = *Gallus gallus*; Hs = *Homo sapiens*; Mm = *Mus musculus*; Tn = *Tetraodon nigriviridis*; Tr = *Takifugu rubripes*; Xt = *Xenopus tropicalis*.

We next examined the relationship of the TSP-4-like sequences in fish to mammalian TSP-4 in more detail. Although the highest BLASTP bit scores are with TSP-4, the sequences also had extensive similarity with TSP-3 and TSP-5, when compared on the basis of their C-terminal regions (Table 2). We examined all the fish subgroup B sequences for the presence or absence of the globular TSP amino-terminal domain. All the predicted fish TSP-3s and many of the TSP-4s contained a TSP amino-terminal domain. However, in each fish genome, one of the TSP-4-like sequences (*T. nigroviridis *TSP-4b, *T. rubripes *TSP-4b and *D. rerio *TSP-4b, respectively) lacked the amino-terminal domain [see Additional File 1]. This finding opened up the possibility that, despite their overall highest sequence identity with mammalian TSP-4 polypeptides, these proteins are related to tetrapod TSP-5/COMP. To further examine the relationships of fish TSP-4s to tetrapod TSP-4 and TSP-5, the highly-conserved C-terminal regions, (i.e., the type 3 repeats and L-lectin domain; [11]), of all the sequences in our dataset were aligned using CLUSTALW and compared as an Phylip unrooted tree. The TSP-1, TSP-2 and TSP-3 sequences each formed a distinct branch in the diagram : i.e., in each case these sequences are more closely related to each other than to any other TSP. In contrast, the TSP-4 and tetrapod TSP-5 sequences formed a broad grouping in which the TSP-5s clustered but were not on a distinct branch in relation to the TSP-4s (Fig. 2A). In similar unrooted trees made without the fish TSPs, the five TSPs of tetrapods each form a separate branch [5]. To evaluate how well supported the TSP-3, TSP-4 and TSP-5 branches are, we also prepared a TCOFFEE alignment and conducted phylogenetic analysis by the PHYML maximum-likelihood algorithm that includes bootstrap analysis (Fig. 2B). Both analysis methods consistently strongly supported the key branches leading to the TSP-1 and TSP-2 groups and the TSP-3 group as forming a distinct sub-branch. However, the PHYML analysis produced a different ordering of the branches leading to the TSP-3, TSP-4 and TSP-5/COMP groups and the bootstrap analysis indicated only weak support for nodes relating to the TSP-4 and TSP-5 sequences (Fig. 2B). Thus, the molecular phylogenies suggested a possible close relationship between TSP-4 and TSP-5, but did not provide a clear resolution of the relationships of the TSP-3, TSP-5 and TSP-5/COMP sequences.

**Table 2:** Relationships of fish TSP-4-like sequences to human TSP-3, TSP-4, or TSP-4.

	% Identity to Human :		
	
TSP C terminal region of:	TSP-3	TSP-4	TSP-5
*T.rubripes *TSP-4a	78	81	79
*T.rubripes *TSP-4b	67	72	70
			
*T.nigroviridis *TSP-4a	75	80	75
*T.nigroviridis *TSP-4b	75	80	78
*T.nigroviridis *TSP-4c	72	80	73
			
*D.rerio *TSP-4a	72	78	75
*D.rerio *TSP-4b	75	81	79
			
*M.musculus *TSP-4	78	96	80
*M.musculus *TSP-5	73	80	95
			
*H.sapiens *TSP-4	78	100	80
*H.sapiens *TSP-5	73	80	100

% identity values are on the basis of the C-terminal regions (type 3 repeats and L-lectin-like domain; equivalent of human TSP-4 aa 443–916). Mammalian TSP-4 and TSP-5 are shown for comparison.

### Syntenic relationships of tetrapod and fish TSP genes : TSP-5/COMP is encoded at an ancient locus

The species-specific encoding of paralogous pairs of TSP -1-, TSP-3-, or TSP-4 in fish raised the possibility that these TSP genes exist as a result of the additional genome duplication that took place early in the Actinopterygii (ray-finned fish) lineage [36,45,46]. In addition, the intriguing possible relationship between fish TSP-4-like sequences and TSP-5 suggested that tetrapod TSP-5/COMP might have arisen through a relatively recent gene duplication of TSP-4 with subsequent loss of the exons encoding the amino-terminal domain. If TSP-5/COMP did arise from a recent TSP-4 gene duplication then, according to the molecular clock hypothesis, the encoded protein would be expected to have closer sequence identity to TSP-4 than to other members of subgroup B [47]. Our molecular phylogenies (Fig. 2) and other phylogenetic studies have not convincingly resolved the relationships of tetrapod TSP-3, TSP-4 and TSP-5 [48,49]. The overall pairwise sequence identities of TSP-3, TSP-4, and TSP-5 are very similar in any given tetrapod species. For example, in pairwise comparisons of the region from the coiled-coil domain to the C-terminus of human subgroup B TSPs, the identity between TSP-3 and TSP-4 is 60 %, between TSP-3 and TSP-5 is 58 %, and between TSP-4 and TSP-5 is 63 %. Similar results are obtained if the comparison is made in other tetrapod species (data not shown). Furthermore, the exon organization of the TSP-3, TSP-4 and TSP-5 genes in human and mouse are near-identical, with the TSP-5/COMP gene lacking the four exons that encode the amino-terminal domain [50-52]. Therefore, as an independent approach to understand the evolutionary relationships between fish and tetrapod TSPs, in particular the relationship of TSP-4 and TSP-5, we undertook a phylogenomic analysis of the conservation of neighboring genes around each TSP gene locus in the available mapped fish and tetrapod genomes. Conservation of synteny is a powerful approach to reconstruct evolutionary processes when multiple physically-mapped genome sequences are available. The criterion for conservation of synteny is that orthologous gene loci are linked in different species, irrespective of the exact gene order or the presence of non-conserved intervening genes [53].

**Figure 3:**
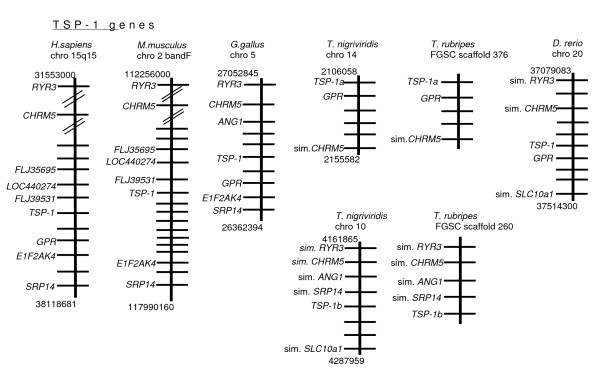
**Syntenic relationships of TSP-1 gene loci in fish and tetrapods**. The physically-mapped genomes of human, mouse, chicken, *T. nigriviridis*, *T. rubripes *and *D. rerio *were used to identify conserved gene neighbors of the TSP-1 gene. In each panel, each diagram represents the order of genes on the chromosome in the vicinity of the relevant TSP gene. Each horizontal line represents a gene; only the conserved genes are labeled with gene names. Protein designations are used for genes lacking a gene name. TSP genes are shown in bold. Numbers above and below each diagram refer to the position on the chromosome in bases. For visual simplicity of presentation, all diagrams are orientated for similarity of gene order.

First, we examined the NCBI mapped genomic scaffolds to identify genes immediately adjacent to the TSP-encoding loci of human, mouse and chicken, because TSP-1 to -5 were originally defined in these species. For each TSP gene, we could identify local neighboring genes that have been conserved between all three species. In the case of the TSP-1 gene, the RYR3, *CHRM5, E1F2AK4 *and *SRP14 *genes were syntenic with the TSP-1 gene in all three species and several other genes (*GPR, FLJ39531 *and *FLJ35695*) were conserved between two species (Fig. 3). These conserved neighboring genes provided a "fingerprint" by which to recognize the orthologous TSP-1 locus in other species. We found that that the *GPR *and *CHRM5 *genes are conserved in the vicinity of the TSP-1a genes of *T. nigriviridis *and *T. rubripes *and the single TSP-1 gene of *D. rerio*. The *RYR3 *gene was also conserved adjacent to *D. rerio *TSP-1 (Fig. 3). *RYR3, CHRM5 *and *SRP14 *were also adjacent to the TSP-1b genes of *T. nigriviridis *and *T. rubripes*, providing clear evidence that both TSP-1 genes in pufferfish are paralogues that arose through duplication of an ancestral TSP-1-encoding locus that was common to fish and tetrapods. This intepretation is also supported by the presence in *T. rubripes *of *ANG-1 *that is also adjacent to the chicken TSP-1 gene (Fig. 3).

**Figure 4:**
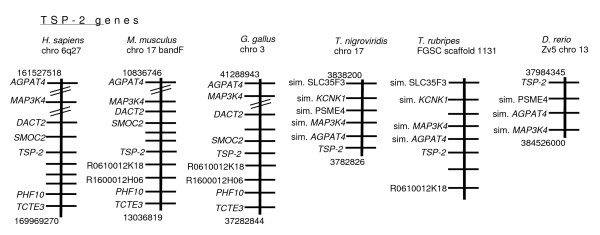
**Syntenic relationships of TSP-2 gene loci in fish and tetrapods**. The physically-mapped genomes of human, mouse, chicken, *T. nigriviridis*, *T. rubripes *and *D. rerio *were used to identify conserved gene neighbors of the TSP-2 gene. Diagrams are arranged as in Fig. 3.

For the TSP-2 gene, six neighboring genes (*AGPAT*, *MAP3K4, DACT2*, *SMOC2, PHF10 *and *TCTE3*) are conserved between human, mouse and chicken. Loci encoding RO610012K18 and R1600012H06 are also conserved between mouse and chicken (Fig. 4). *AGPAT4 *and *MAP3K4 *are conserved in all three fish species and the gene encoding RO610012K18 is also conserved in *T. rubripes*. Additionally, *SLC35F3 *and *KCNK1 *are adjacent in both pufferfish species : these genes are syntenic with TSP-2 in chicken but not in mouse or human (Fig. 4 and data not shown).

**Figure 5:**
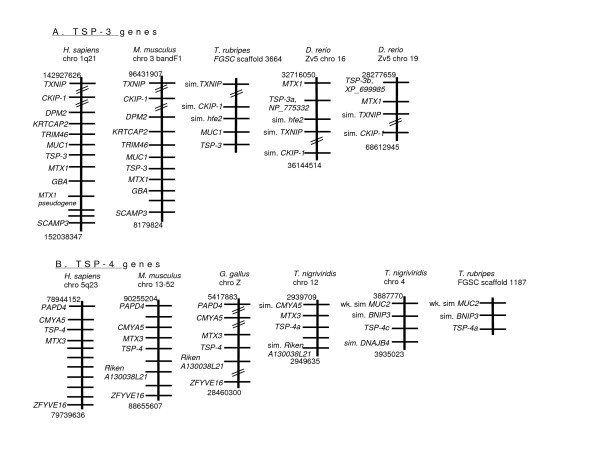
**Syntenic relationships of TSP-3 and TSP-4 gene loci in fish and tetrapods**. The physically-mapped genomes of human, mouse, chicken, *T. nigriviridis*, *T. rubripes *and *D. rerio *were used to identify conserved gene neighbors of the TSP-3 gene (panel A) and the TSP-4 gene (panel B). Diagrams are arranged as in Fig. 3. *D. rerio *TSP-4a is not included because sequence assembly for this region is unfinished in Zv5 (Wellcome Trust Sanger Institute).

The TSP-3 genes of human and mouse are part of a well-conserved gene cluster that includes the genes encoding metaxin-1 (*MTX1*) and the polymorphic epithelial mucin (*MUC-1*) (Fig. 5A). In human and mouse, the TSP-3 gene shares a common promoter region with *MTX1 *and is transcribed divergently. An adjacent metaxin pseudogene has also been recognized [54,55]. Other genes local to the TSP-3 gene (*TXNIP1, CKIP-1, DPM2, KRTCAP2, TRIM46, GBA *and *SCAMP3*) were also conserved between human and mouse. Although expression of the chicken TSP-3 transcript has been well-characterized, [56], the chicken TSP-3 gene is as yet unmapped and was therefore unavailable for comparison. All the fish TSP-3 gene loci were syntenic with the tetrapod TSP-3 genes, on the basis of conservation of at least two of the adjacent genes (Fig. 5A: because the TSP-3 gene of *T. rubripes *is located at the end of the scaffold sequence the presence of *MTX1 *could not be assessed). The conservation of similar neighboring genes identified *D. rerio *TSP-3a and TSP-3b as paralogues that arose through duplication of an ancestral TSP-3-encoding locus.

Interestingly, the TSP-4 genes of human, mouse, and chicken are all immediately adjacent to the gene encoding another member of the metaxin family, metaxin-3 (*MTX3*). Three other flanking genes are conserved between human, mouse and chicken, *CMYA5, PAPD4 *and *ZFYVE16*. The gene encoding Riken A130038L21 is also conserved adjacent to the mouse and chicken TSP-4 genes (Fig. 5B). Of the TSP-4-like genes of fish, TSP-4a in *T. nigriviridis *is encoded adjacent to *MTX3, CMYA5 *and A130038L21 and was thus established as syntenic with tetrapod the TSP-4 gene (Fig. 5B). Genes adjacent to *T. rubripes *TSP-4a did not include *MTX3 *but were similar to those adjacent to *T. nigriviridis *TSP-4c (discussed further below).

**Figure 6:**
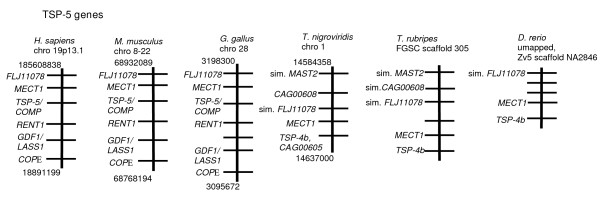
**Syntenic relationships of TSP-5 gene loci in fish and tetrapods**. The physically-mapped genomes of human, mouse, chicken, *T. nigriviridis*, *T. rubripes *and *D. rerio *were used to identify conserved gene neighbors of the TSP-5 gene and to identify orthologues of the TSP-5 gene in fish. Diagrams are arranged as in Fig. 3.

With regard to the other fish genes encoding TSP-4-like proteins, we first examined the chromosomal region of the tetrapod TSP-5/COMP genes. In human, mouse and chicken the TSP-5 gene has a distinct set of conserved gene neighbors, *FLJ11078, MECT1, RENT1, GDF1/LASS1 *and *COP*E (Fig. 6). With these clear criteria for identification of the TSP-5 gene in hand, one TSP-4-like encoding sequence in each fish genome (TSP-4b, CAG00605, of *T. nigriviridis*; TSP-4b, scaffold 305, of *T. rubripes*, and TSP-4b, XP_690679, of *D. rerio*) was found to be encoded at a locus syntenic with tetrapod TSP-5/COMP (Fig. 6). These data define that the gene that encodes TSP-5/COMP in tetrapods predates the divergence of fish and tetrapods.

In *T. nigroviridis*, the TSP-4c gene has gene neighbors unrelated to those of TSP-4 or TSP-5. The same gene neighbors were conserved adjacent to *T. rubripes *TSP-4a (Fig. 5B). We infer that the fish-specific duplication of the TSP-4 gene was accompanied in the puffer-fish lineage by transposition of one of the duplicated genes. Both paralogues have been retained in *T. nigriviridis *whereas the TSP-4 gene at the ancestral locus has been lost in *T. rubripes*.

### Evidence for paralogous relationships between four TSP-encoding loci in the human genome

The above results clarified the identities of fish TSP genes in relation to tetrapod TSP genes, yet still did not resolve certain ambiguities with regard to the relationships of the TSP-3, TSP-4 and TSP-5 genes. At the level of genome organization, the conserved synteny of both the TSP-3 and TSP-4 genes with genes encoding members of the metaxin family suggests that the TSP-3 and TSP-4 genes lie within paralogous genomic regions that arose from the same ancestral DNA duplication event [57]. On this basis, the TSP-3 and TSP-4 genes can be considered closely related. Because no metaxin gene is found adjacent to the TSP-5/COMP locus, or indeed on the same chromosome in any of the organisms studied, and other local conserved gene neighbors of the TSP-5/COMP gene are distinct from those conserved adjacent to the TSP-4 gene (Fig. 5B and Fig. 6), the TSP-5 gene appears more remote from TSP-3 and TSP-4. Yet, by criteria of protein sequence relationships, the new data from fish demonstrate a very close relationship between TSP-4-like coding sequences and TSP-5 (Fig. 2). To integrate these separate and apparently paradoxical pieces of data, we took advantage of the extensive analysis of human genome sequence organization that has identified large paralogous chromosomal regions within the human genome itself. The existence of such regions provides evidence for the rapid evolution of vertebrate genomes through large-scale block or genome-wide DNA duplication in an ancestral chordate [57-59]. We tested whether any of the five TSP-encoding loci are located in paralogous region of the human genome by searching the "dataset of paralogons in the human genome", version 5.28 [57]. The human genome is suitable for this form of analysis because the rate of DNA rearrangement has been slower than in rodents [60].

**Figure 7:**
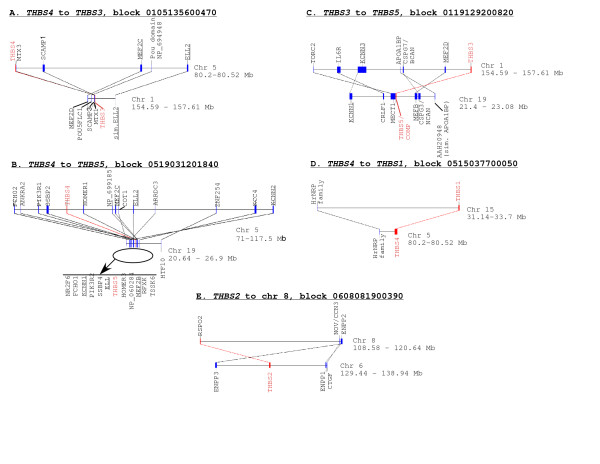
**Paralogous relationships of TSP-encoding loci in the human genome**. The database of Paralogons in the human genome, version 5.28, was searched for evidence of large-scale similarities of genomic organization in the chromosomal regions of the five thrombospondin genes. Panels A to E show the five paralogous blocks identified, their chromosomal locations, and the gene pairs that make up each block. The genomic regions of subgroup B TSPs are strongly related, the TSP-1 and TSP-4 loci are marginally related and the TSP-2 gene is located within an unrelated region of the genome. In each block, the paired TSP genes are labeled in red. Other genes present in multiple blocks are underlined. Genes are identified according to HUGO gene nomenclature where available, or by the GenBank accession number of the encoded predicted protein. In B, gene order on chromosome 19 is displayed in an expanded view.

The TSP-4 gene at 5q23 was located within a chromosomal block with significant paralogy (6 pairs of shared genes) to the chromosomal block of the TSP-3 gene (Fig. 7A). Importantly, the TSP-5/COMP locus at chromosome 19p13.1 was identified to lie within a chromosomal region with clear paralogy to a block of chromosome 5 that included the TSP-4 gene (13 pairs of shared genes; Fig. 7B). Although located within a 5 Mb region of chromosome 19, the paralogous genes are spread throughout a 46.5 Mb region of chromosome 5, explaining why the relationship was not detected by analysis of local neighboring genes. The TSP-5/COMP locus is also paralogous with the region of the TSP-3 gene on chromosome 1q (7 pairs of shared genes; Fig. 7C). Interestingly, paralogy of the TSP-4 region to the TSP-1 locus at 15q15 was also detected, albeit on the basis of two pairs of related genes (Fig. 7D). The TSP-2 locus at 6q27 was not paralogous to any of these regions but was part of a separate block of paralogy with a region of chromosome 8 (4 pairs of shared genes; Fig. 7E). We infer that the TSP-2 gene underwent replicative transposition subsequent to the duplication event that gave rise to the TSP-1 and TSP-2 genes.

To substantiate these findings, additional paralogy searches were carried out for the three members of the metaxin family: the searches with metaxin-1 and metaxin-3 again identified the paralogy between the chromosomal regions of TSP-3 and TSP-4. No paralogous region was identified with regard to the metaxin-2 locus on chromosome 2 (data not shown). Of the other gene pairs identified within the paralogous regions of the TSP-3, TSP-4 and TSP-5 genes, members of the *MEF *(myocyte enhancer factor) and *KCNN *(potassium intermediate/small conductance calcium-activated channel, subfamily N) families were consistently present in all the paired blocks (Fig. 7A–C; KCNN paralogy is not shown in Fig. 7A, but *KCNN2 *is located at 113.73 Mb of chromosome 5 and *KCNN3 *is located at 151.7Mb of chromosome 1; [61]. Thus, the ancestral chromosomal region likely included ancestral MEF and KCNN genes in the vicinity of a TSP gene. We tested this idea by examining whether MEF or KCNN family members are also syntenic with TSPs in other vertebrates. From the available mapping information, *MEF-2D *in mouse and zebrafish are located on the same chromosomes as the TSP-3 gene (TSP-3a on chromosome 16 in the case of zebrafish), and *MEF-2C *and *MEF-2B *in the mouse are located on the same chromosomes as TSP-4 and TSP-5, respectively. *KCNN1 *is also on mouse chromosome 8; *KCNN2 *is syntenic with TSP-4 in chicken but not in mouse, and *KCNN3 *is syntenic with TSP-4b (i.e. the TSP-5 locus) in *T. nigriviridis*. These data reinforce the intepretation that the TSP-3, TSP-4 and TSP-5 genes have evolved as a consequence of duplications of the same ancestral genomic region.

## Discussion

Our study, initiated with the aim of assessing the suitability of zebrafish as a model organism for future experimental study of TSPs in relation to their roles in human disease, delivers some unexpected conclusions that change current perspectives on the TSP gene family in vertebrates. Based on a combination of molecular phylogenetic and phylogenomic approaches, we propose a new model for the evolution of TSPs in vertebrates.

The encoding of large numbers of TSPs in three species of fish, that include paralogous pairs of TSP-1, TSP-3, or TSP-4 genes, is in line with the strong evidence that ray-finned fish underwent an additional whole genome duplication after the divergence of the bony fish and tetrapod lineages around 450 million years ago [36,45-47]. In general, after a gene duplication event, reduced selection pressure on one of the paralogous genes can have several consequences. One gene may be lost relatively rapidly, or both genes may be retained and diverge functionally, either by sub-specialization of the original function or by evolving new functions [62,63]. For the TSP family, the three fish species provide evidence of distinct lineage-specific events involving loss or retention of different TSP paralogues. For example, *T. nigriviridis *encodes two TSP-1s but does not encode a TSP-3, whereas *D. rerio *encodes two TSP-3s and a single TSP-1 (Table 1). The retention of both members of a paralogous pair may have resulted in functional specialization. Thus, each of the fish TSP-1 or TSP-3 paralogues could have a subset of the functions of tetrapod TSP-1 or TSP-3, or may have evolved distinct and novel functions.

We could readily identify synteny of the TSP-encoding loci in fish with the chromosomal regions of tetrapod TSP genes. This finding establishes that precursors of the TSP-1 to TSP-5 genes were all present within corresponding ancestral genomic contexts in the last common ancestor of bony fish and tetrapods. This state appears to have originated within the chordate lineage. The *Ciona intestinalis *(an invertebrate chordate) genome encodes a smaller number of TSPs; yet, because both A and B forms of TSPs are present, it is clear that the existence of A and B forms predates the whole genome duplications that occurred in the early stages of vertebrate evolution ([5,64]; our unpublished data). These conclusions are supported by evidence that large scale gene duplication activity increased substantially after the divergence of amphioxus (a cephalochordate) from the vertebrate lineage [65]. Whereas *Ciona intestinalis *encodes a single subgroup A TSP (GenBank AAS45620; [5]), inspection of available ESTs from a cartilaginous fish, the little skate *Leucoraja erinacea*, indicates that transcripts corresponding to both TSP-1 and TSP-2 are present (GenBank CV068535 and CV067510). Thus, for subgroup A, an expansion of gene number appears common to both cartilaginous and bony fish. This observation is in agreement with a recent statistical estimate that most vertebrate-specific gene duplications occurred before the separation of cartilaginous and bony fish [66]. For additional clarification of the phasing of expansion of the TSP gene family in the chordate and vertebrate lineages, the genome sequences of a jawless vertebrate (i.e., lamphrey or hagfish) and a cephalochordate are needed.

**Figure 8:**
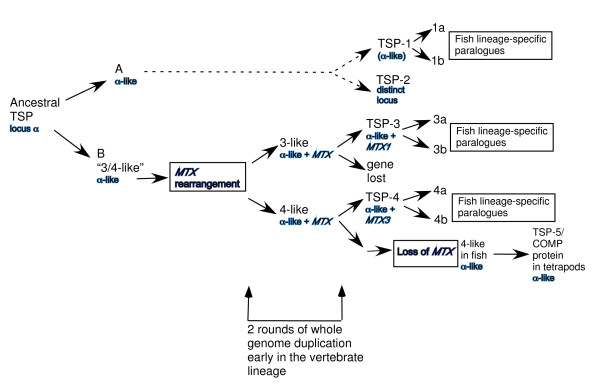
**Model for the evolution of vertebrate TSPs, based on evidence of protein sequence phylogeny, conserved synteny of genomic loci between species, and paralogous relationships between the genomic regions of human TSPs**. The diagram also takes into account that the A and B forms predate the whole genome duplications that occurred early in the vertebrate lineage [5]. TSP genes and proteins are indicated in black and their genomic contexts in blue. Dotted line indicates that intermediate steps are not represented for TSP-1 and TSP-2. See Discussion for details.

A second major finding from the phylogenomic analysis was the definition of the conservation of the TSP-5/COMP-encoding locus. Although the overall sequence characteristics of the TSP-5/COMP protein appear specific to tetrapods, the encoding locus is common to both bony fish and tetrapods (Fig. 6). Thus, the TSP gene at this locus did not originate in tetrapods. In fish, the similarity of the encoded protein sequence to TSP-4 suggests that the gene arose through duplication of an ancestral TSP-4-like gene, with subsequent loss of the exons encoding the amino-terminal domain. This view is strongly supported by the clear large-scale paralogy between the chromosomal regions of the human TSP-4 and TSP-5 genes. However, whereas all vertebrate TSP-3 and TSP-4 genes are encoded adjacent to a metaxin family member, no metaxin gene is present on the same chromosome as the TSP-5/COMP gene in any genome. The most parsimonious intepretation of these data would be that, subsequent to an initial duplication of a TSP-4-like gene, an ancestral metaxin gene became transposed adjacent to one of the paralogues. Reduplication of this region then gave rise to TSP-3 and TSP-4, adjacent to metaxin-1 and metaxin-3, respectively. However, this scenario puts the TSP-4-like/TSP-5 gene duplication before the TSP-4/TSP-3 gene duplication. This appears unlikely in view of : 1), the high identity of the polypeptide encoded by the fish TSP-5 gene to TSP-4, suggestive of a recent relationship; 2), similarly, paralogy between the genomic contexts of the human TSP-4 and TSP-5 genes is stronger than that between TSP-3 and TSP-4; 3), the presence of a TSP-3-like TSP in the basal chordate, *Ciona intestinalis*, (*Ciona *TSP-B [5], Gene Cluster 13925 [68]). Taking the genomic context and protein sequence evidence together, a new model for the evolution of TSPs in vertebrates is proposed (Fig. 8).

Our studies also lead to the novel and surprising conclusion that the TSP-5/COMP protein sequence has evolved to its current state as an innovation of tetrapods. In human, mouse, chicken and *X. tropicalis*, TSP-4 and TSP-5 protein sequences are readily distinguished by BLAST searches or multiple sequence alignment, even without consideration of the presence or absence of the TSP amino-terminal domain (e.g. [5,11]; Table 2). In contrast, in fish, the proteins encoded at the TSP-5/COMP locus have sequence character most similar to TSP-4, even when the full-length sequence is used as the BLASTP query. None of the invertebrate TSPs identified to date has TSP-5 character ([5,6,68]; our unpublished observations). Thus, on the basis that the TSP-5 locus arose through duplication of an ancestral TSP-4-like gene, it appears that the encoded protein retained TSP-4-like character in fish and has evolved distinct and novel features in tetrapods. Given the significant role of TSP-5/COMP in mammalian cartilage, it is tempting to speculate that the polypeptide sequence evolved rapidly in tetrapods under the altered selection pressures imposed on the bony endoskeleton by the switch from aquatic swimming to terrestrial locomotion. Although it has been accepted that TSP-4 and TSP-5 have separate biological activities in mammals, there are interesting hints of over-lap. For example, both TSP-4 and TSP-5 are expressed in blood vessel walls [69,70]. In chick embryos, TSP-4 is transiently expressed in cartilage in association with the initial stages of osteogenesis [71]. Further consideration of similarities and differences in the characteristics, regulation, and pathologies of TSP-4 and TSP-5 may open fruitful novel directions for future research.

## Conclusion

Combining the approaches of molecular phylogeny and phylogenomic analysis of chromosomal context is a generally applicable strategy to improve the identification of orthologous relationships between members of complex gene families across species. The identification of numerous fish TSPs and the discovery of the unexpectedly close relationship between TSP-4 and TSP-5 raise fascinating questions about the fundamental roles of TSPs in fish. New directions are identified for studies of the pathophysiological roles of TSP-4 and TSP-5 in human disease.

## Methods

### Dataset of known vertebrate TSPs

The following TSP protein sequences, predicted from sequencing of full-length cDNAs, were included in our studies : from *Homo sapiens*, TSP-1 (GenBank Accession P07996); TSP-2 (P35442); TSP-3 (P49746); TSP-4 (P35443) and TSP-5/COMP (P49747); from *Mus musculus*, TSP-1 (A40558); TSP-2 (Q03350); TSP-3 (U16175); TSP-4 (AF152393); TSP-5/COMP (AF033530); from *Gallus gallus*, TSP-2 (L81165; 72), and from *Danio rerio*, TSP-3 and TSP-4 (NP_775332 and NP_775333; [42]). Partial sequences predicted from cDNA included *G. gallus *TSP-1 (U76994; [56]), TSP-3 (L81165; [56]) and TSP-4 (L27263; [71]).

### Identification of novel TSPS in fully-sequenced genomes of vertebrates and from expressed sequence tags

Human TSP-1 and TSP-5 were used as the query sequences in TBLASTX or BLASTP searches carried out at NCBI and UCSC Genome Bioinformatics portals against the fully-sequenced genomes and, as available, the genome-predicted proteins of the fish *Takifugu rubripes *([36]; assembly 3 with 5.7× coverage); *Tetraodon nigroviridis *([37]; assembly 1.1 with 8.3× coverage); *Danio rerio *([38] and from August 2005, Zv5 with 5–7× coverage [39]); the amphibian *Xenopus tropicalis*, ([40]; assembly 4.1, 7.65× coverage, searched via DOE Joint Genome Institute), and the bird *Gallus gallus *([35]; assembly 1 with 6.6× coverage). Accession and scaffold numbers used in this article are as of October 2005. Each matching sequence returned with an expectation value less than e= 0.0001 was used to query the GenBank non-redundant protein database, to establish the assignment as a TSP and to identify which of the mammalian TSPs 1–5 had the closest sequence identity. *X. tropicalis *sequences were also compared with available sequencs from *Xenopus laevis : *TSP-1 (P3544); TSP-3 (AAH48222) and TSP-4 (Z19091) [73]. Sequences were also searched by TBLASTX against dbEST (database of expressed sequence tags) at NCBI for ESTs from the corresponding organism, to establish the existence of transcribed sequences corresponding to the open reading frame predicted from genomic DNA. In some cases, EST sequences and comparisons with known TSPs were used to extend or correct the genome-predicted sequences. Searches of dbEST for TSP ESTs in other fish species were carried out by limiting the query to the Entrez criteria *Chondrichthyes *or *Teleostomi*. Taxonomic classifications were based on the Tree of Life Project [74].

### Analysis of domain architecture and oligomerization potential of novel TSPs

The domain architecture of the predicted novel TSP proteins was evaluated by searches against the Conserved Domain Database (CDD) database at NCBI [75], the Simple Modular architecture research tool (SMART) domain database at EMBL [76], and the InterPro database [77] via ExPasy [78], supplemented by manual inspection. Sequences were assigned to TSP sub-group A if they contained a vWF-C domain and TSP type 1 repeats and to TSP subgroup B if these domains were not present and the sequence included additional EGF-like domains [17]. Sequences were analyzed for the presence of a coiled-coil region using the program COILS [79]. Although most sequences in our set covered full-length TSPs, *G. gallus *TSP-3 is at present identified only as a partial cDNA that does not include the coiled-coils [56].

### Multiple sequence alignment and phylogenetic trees

Multiple sequence alignments of the coiled-coil domains were prepared in TCOFFEE, that combines pairwise/global and local alignment methods into a single model [80]. Alignments of the sixth type 3 repeat or the C-terminal region (i.e. the type 3 repeats and L-lectin domain) were prepared by the progressive, neighborhood-joining alignment method, CLUSTALW [81]. The C-terminal region was also aligned by the TCOFFEE algorithm. The multiple sequence alignments are presented in Boxshade 3.2. For preparation of phylogenetic trees, gaps due to variations present in less than 10 % of the sequences were removed from the alignments. Unrooted trees were constructed either from the Phylip distance matrix output of the alignments in DRAWTREE, using UCSD Biology workbench 3 tools [82], or by the maximum-likelihood method, PHYML, using the WAG substitution model and 100 bootstrap cycles [83]. Unrooted trees are presented in D.G. Gilbert's Phylodendron, version 0.8d [84].

### Identification of syntenic relationships

The chromosomal locations of TSP-encoding genes were identified by TBLASTN searches of the physically-mapped genomes of the human (build 35.1) [61], mouse (build 34.1), [85], and chicken (build 1.1) [41] through the BLAST Genomes interface at NCBI, using in each case the TSP protein sequences encoded within the genome of interest as the queries. For each TSP gene in human, mouse and chicken, local syntenic genes were identified using the map viewer and Genemap Tables at NCBI. In the case of *Tetraodon nigroviridis*, positions of TSP-encoding genes were identified within the Genoscope physically-mapped shotgun scaffold sequences. This permitted their assignment to a chromosome and identification of the GenBank accession numbers for the neighboring predicted protein-coding sequences. The identification of each predicted protein was then accomplished by BLASTP searches of GenBank. Genomic locations and gene neighbors were also analyzed by BLAT search of the genome at UCSC Genome Bioinformatics. In the case of *Takifugu rubripes*, the predicted TSP protein sequences were mapped onto the genomic scaffolds by TBLASTN searches. Adjacent coding sequences on the scaffold were then identified by BLASTX searches of GenBank proteins and by viewing of genome-predicted proteins on the genome contigs at UCSC Genome Bioinformatics. In the case of *D. rerio*, initial identification of gene neighbors was made from the NCBI Genemap Table of the 2004 Zv4 assembly. Gene neighbors were re-confirmed on the contigs of the 2005 scaffold assembly Zv5 at Ensembl (EBI) [39]. For identification of parologous TSP-encoding regions in the human genome, the database of "Paralogons in the human genome", version 5.28, was searched [57]. In the figures, genes encoding known proteins are identified according to HUGO gene names where available. GenBank gene locus numbers, or accession numbers of the encoded proteins, are given for previously unknown genes. Because TSPs have not yet been assigned gene symbols in all the species studied here, they are all designated TSP-1, TSP-2, etc, in Figs. 3, 4, 5, 6.

## Abbreviations

BLAST, basic local alignment search tool; COMP, cartilage oligomeric matrix protein; ECM, extracellular matrix; EGF, epidermal growth factor; EST, expressed sequence tag; OMIM, Online Mendelian inheritance in man; TSP, thrombospondin.

## Authors' contributions

PM, SC and JB conducted the searches for TSPs in three fish genomes. PM analyzed additional fish genomes, the *X. tropicalis *genome, and dbest. SC and JB analyzed TSP domain architectures and motifs. JCA analyzed synteny and paralogy and completed the figures and the writing of the paper. All authors contributed text to drafts of the paper and all approved the final version.

## Supplementary Material

Additional File 1 The file contains the amino-terminal domains of the vertebrate TSP sequences in the dataset. The coiled-coil regions are highlighted in yellow. Click here for file
