# Supplementary material for: Phylogenomic analysis of vertebrate thrombospondins reveals fish-specific paralogues, ancestral gene relationships and a tetrapod innovation
Source: BMC Evol Biol. 2006 Apr 18;6:33. doi: 10.1186/1471-2148-6-33 (PMC1464143; doi:10.1186/1471-2148-6-33)
Supplement: Additional File 1 — The file contains the amino-terminal domains of the vertebrate TSP sequences in the dataset. The coiled-coil regions are highlighted in yellow. [file 1471-2148-6-33-S1.rtf]

ADDITIONAL  DATA FILE 1. 
The amino-terminal domains of vertebrate TSP sequences in the dataset. The coiled-coil regions are highlighted in yellow.  


>TnTSP-1a
GIFLLLILWTCESTRVAESRDDNSVYDLFELVQVPRKNHGVTLVKGDDPYSPAYKILDPDLIPAVPDQAFSDLIDSIRAERGFLLLLNFKQFKRTRGSLLTVEKKDGSGPVFEIISNGKANTLDIVFSTENKQQVVSIEEEDQAQLYVGCEDVNTAELDAPIQSILTQETPAGARLRIGKGAVNDRFMGVLQNVRFVFGTTLDAILRNKGCQNSISSETMILENLNGSSAIRTEYTGHKTKDLQMVCGFSCEDLFSMFKELKLGVVVKELSNELRQLTDENKLIKNHIGIHNGVC

>TnTSP-1b
MMLCGIFLLLMLWGCEGARLAGSPDDNSVYDLFDLARVSKRQNGVTLVKGADPYSPAYKVLNADLIPSVPDSSFRDILDSILAERGFLLIINLKQFRKSRGSILTIEKNDGSGSIFEIVSNGRANTLEVLYSTANHQQVVTIEDTDLATGHWKNITLFVQDDRVKLYVGCEEVNEAEMDFPIHKVLTQELADVATLRIAKGAVRDKFTGVLQNVRFVFGTSLDEILRNKGCQNGAVLTDVMTLENPVNGSSPAVQTDHPGHKAKDVQPGQVCGLSCEDIAGIFKELRGLGVVVRKLSIDLRKVSEESMLLKNETKQSGIC

>TnTSP-2
GEQENETSFDLFEISHITRRTLGAKQFRGPNSDTPAYRFIRFDHLPPVSTPILKQILRQMQYNEGFVFMASMRQDRTSRGTLIGFEGPNGQRQFEIVSNGRANSLDLVYSVDGSQNIVQFEEVDLSDSQWKNITLHVHGENANLFVGCSLIDSFILDEPFYEHLQAEGSRMYVAKGSSRESHFRGLLQNVRFMFDTTVEDILLSRDCEVPKQDDGNIVRESTEIMDVSPSITTNVIGQKTDEVGSNMCERSCEELSTMFQELNGLRVVVSNLIDGLQKVTEENMFMKEALGKMKNS

>TnTSP-4a
MMRVWAKAAALTLLLQQLVLTVAAQGIVYDLLDSPDCLPDLLQGSLKNKGRDEAFLLSSFRLQSRAPTSLYSVVNPKDSSKYLELSVQAKLSKASTTAPLADGRDHHMMLHASGLQAVPPRLNIYIDCRLVHTMNDLPAAFWGSPAGSQHGGAQDPAHSRTGERVQQALFFYKSVHRLAGLCLYFPFEDKLTDLKLVVEDTVDNVATLQDCHMDQGQPLQLLEIQGPRMVHDQATVQELKSMFAEMKELLQQQIKETNFLRNTIAECLAC
 
>TnTSP-4b
MLGFLLLSCVLHTGGHAAAAPRDGEIISQIKLTNIALAEIKELLKQQVNSHIKEITFLKNTVMECEAC

>TnTSP4c
MGVWTALVVALQLLLKPCLRVEAQVLGERRIHRLSANDSVYDLLTSPDCLPDLLQGGLVEQGVNEAFILTSFKLQPKTGTSVFGLYNPRDNSKYFEFTVMGKLNRAVLRYLRSDKRMSSVTFNNLGLPAGYGLVELRTMQSRDQDSLDELKLLVGDSFENVASLQGCHFQQKDSVQTLGVNTKQLSNQMLELTKVINELKDVLIQQVKETSFLRNTISECQAC

>TrTSP-1a
MKLTGIFLLLILWTCECTREAESRDDNSVYDLFELVQVPRKNHGVTLVKGDDPYSPAYKILNPDLIPAVPDNAFSDLIDSIHAERGFILMLNFKQFKRTRGSLLTVEKKDGSGPVFEVISNGKANTLDIVFSTENKQQVVSIEDVDLATSHWKNITLFVQEDQAQLYVGCEDVNTAELDAPIHSILTQETPASARLRIGKGAVNDRFMGVLQNVRFVFGTTLDAILRNKGCQNSVTSETMILENLNGSSAIRTEYTGHKTKDLQMVCGFSCEDLISMFKELKSLGVVVKELSNELRQLTDENKLIKNRIGIHNGV

>TrTSP-1b
MMLCGIFLLLMLSGCGGARLAGSADDNSVYDLFDLARVSKRQNGVTLVKGADPYSPAYKVLNADLIPSVPDSSFRDILDSILAERGFLLIINLKQFKRSRGSILTIEKNDGSGSVFEIVSNGRANSLDVVYSIANQQQIASIEDADLATGHWKNITLFVQDDRVKLYVGCEEVNEAEMDLPIHKVLSQDLADIATLRIAKGAVRDKFTGVLQNVRFVFGTSLDAILRNKGCQNGAMLTDVMTLDNPINGSSPSVGSDYTGQKPKDVQPVCGLSCEEISSMFRELRGIGVVVKRLSIDLRKVSEESMLLKNQMNSQSGI

>TrTSP-2
MQNNEGFVFVASIRQDRASRGTLIGFEGPDGQRQFEIVSNGRANSLDLVYWVDGSQNMVQFEDVDLSDSQWKNITLQVHGENANLYVGCSLLDSFILDEPFYEHLQAEGSRMYVAKGSSRESHFRGLLQNVRLLFDTTVEDVLLSRDCEVPKQVAFKWVRELAELHVYGNRRVNAAVRGSNDHANIVSESTEIVDVSPSITTNVIGQKTDEVGPDMCERSCEELSTMFQELKGLRVVVGNLIDGLQKVTEENTLMKEALGKMKNS


>TrTSP-3 
GVYSRQDHRKYLELGIMGKINKVVARYLRADGKIHTVNLQNAIVSDGRVHSIILRLGALQRDHINVELYVDCRLADSSQGAPPLVPLPGEVELVEIRHGQKAYNRLQGAVESLRLLLGGTVASAGALNDCPFQGDSTNYNSVRKQKSSLCHAVLSGDHTKALIGQLIIFNQILGELRLDIREQVKEMALIRNSIMECQVCG

>TrTSP-4a
MGVWTALVVALQLLLCLDVEAQVLGECHTCEVPNSDSDDHFEFAVKVYDLITSPDCLPDLLQGGLVEQGVNEAFILTSFKLQPKTGTSVFGLYNPRDNSKYFEFTVMGKLNRAVLRYLRSDKRMSSVTFNNLVLADGQQHRLLFHLKGMQQQGPGGVELHLDCRLVETVRDLPAAFQGLPAGYGLVELKTMQTRDQDSLDELKLVVGDSFENVASLQGCHFQQRDSVQTLGVNTKQLSNQMLELTKVVNELKDVLIQQVKETSFLRNTISECQAC

>TrTSP-4b
MDRYQKDADVYSFSCFCFSDGEIISQIKMTNIALAEIKELLKQQVKEVGFLKNAMMECEAC

>DrTSP-1
SRDDNSVYDLFELVQVPRKNHGVTLVKGDDPYSPAYKILNPDLIPPVPESAFRDLIDSIHAEKGFLLLVNFKQFKRTRGSLLTVEKNDGSGPVFEIVSNGKANTLDIVFSTENKQQVVSIEEADLAVGHWKNITLFVQEDRVQFYVGCEEVNVAELDASIHTILTQEIPGVAKMRIGKGAVKDRFMGVLQNVRFVFGTTLEAILRNKGCQNSAMTDIITLDNPINGSSPAIRTDYTGHKTKDLQMICGFSCEDLAAMFKELKGLGVVVQELSNELRKVTDDKNMLMNQMGIRAGVC

>DrTSP-2
EDESIFDLFKISGISRKTIGAKLFKGHDWDSPAYRFIRFDHIPAVSTPALHQILKQVQNNEGFVFVATMRQDKGSRGTLLGVEGPGGLRQLEIVSNGRANTLDLVYMVKGSQNVVSFEDVDLSDSQWKNISLYVHGENAHLYVGCSLIDSVILDEPFYELLRPEGSQMFVAKGSIRENHFRGLLQNVQFLFDTPIENILRNKGCEIAKPEEVNVVNESTESVSVGTSISTNFIGEKEKIASDVCERSCEELSNMVQELKGLRIIVGNLIDGLQKVTEENTVMKEVLGNMKNI

>DrTSP-3a
MFVWSAQSDKKQDVPVIDVLGLEDVKQTVAAVEKLSLALKTLSDVYVMSTFRLLPKLGGVLLGLYNKQDNKKYLEVAIMSKINKVLVRYVREDGKLHTVNMQSPNVADGRPQSLILRVGGLRREYLSLELYVNCRLADSAQRLPPLVDLPRDAELVEIRNGHKAYARMQGSMDTLKLALGGTVAQAGALTDCPFQGDASSYNIVNGEVNSILGDHTKALIGQLIIFNQILGELREDIREQVKEMSLVRNAILECQMC

>DrTSP-3b
VINVLELHDVRQTAAAIENLSGALQTVGDLYITSTFMLPPKLGGVLFGLYDKQDNKKYLEIAVVGKINKLLVRYLRSDGKAHTVNLQNPALAEGRTQSLILRIGGLRRSHINLELYVNCRLVDSAQGLPSFVGLPSEAESVDVRTGQKSYARIQGLVESVKLALGGSLATAGLLIDCPFQGDSAINNAVVSDINAILGDHTKALIGQLIIFNQIMGELREDIREQTKEMSLIRNTILECQVC

>DrTSP-4a
MAGTMHLLTAVSLILMLSSANAESIVYNLLTSPDCLPDLLHGGLAEQGVTELYILTTFRIQPGTGNTIFSLYNPRDNSKYFEFSVFGKANKAILRYLRRDGRMSAVTFNKLNLADGEKHRLLFHLKGLEVGHPGGFPHSQGALPVPGVELHLDCRLVETLRDLPAVFNGLNNHQAVELKTMQGKAQEGLEELKLAYGDSVENVASLQDCHTQSDSVQALGLNTKQLTTQMLELTKVINELKDVLIQQVKETSFLRNTISECQAC

>Dr4b
MLWSLCFSCYFYLHSALVSAQGISRDGEIIKQIKGTNQELAEIKELLKQQLIQAAKLDVFNPQTEMNSVLQIQEIVFLKNTVMECEAC

>XtTSP-1
MGMMKGIFLLLMLAVPQTYQAAESGNDDNSVFDLFELTGYNRKTGSRKPEGLHLVKGPDPSSPAYRIEDADLIPPLPEVKFQDLLDAIRADRGFILLATLRQAKKSRGALLSVERKDGAGHIFSLISNGRAGTLDLSLSGERKQQVVSVEDALLATGHWKNITLFVQDDRAQLYVGCDKMENAELDVPIQKIFTEDLASKAHLRVAKGGVKDNFQGVLQNVRFVFGTTLEAILRNKGCSSMTNSVITLDNPVNGSSPAIRTNYIGHKTKDLHAVCGISCDDLSKLFAEMKGLRTLVTSLQDQVTKETERNELIAQRVTMTPGA

>XtTSP-2
KEDETNFDLFEISNINKRTIGAKIFRGHESSSPAYRFLRFDHIPPINNEKMKQIIELMQQNEGFILTATLKQDKNNRGTIISIEGPGISNRLFELVSNGRANTLDLIYWVDGSQNVISLEDVDLADSHWKNITVQLLGENFNLYVGCDLIDSFQLEETFYEHLKDDNIKMFLAKGSIRENHFRGLLQDVRLIFDVSVEDVLRKKGCQLTHTGEVNAISEKTEILHLSPMTMTEYVGQKIDKQVDFCDHSCDELGNMFTELTRLRILVNNLLDNLQKVSEENQVLWELIGPNKTL


>XtTSP-3
MARQSRDLLLLLLLSCLVCWARAARQEVHVIDLLTVSESRHMSGVVEKIRSELLAVNDLYFLSTFRLPPKAGGVLFGLYSKKDNTKWLEASIVGKINKVLVRYMREDSKLHAVSLQNANLSDGNMHTMILRVSGFRGDTLSLELYVDCKQVDTSLGLPEMMIIPQFEVESGDIRSGHKAYLRMQGSVESMKLILGGSLSRVGALSECPFQEDESIHNTVNGVVNSILGEHTKALIAQMTLFNKVLAELREDIRDQVKEMSLIRNTIMECQVC

>XtTSP-4
MPRRKGLCLFLQMLLLHLYGVCQAQPNYQVFDLLAASVQRQVTPFLQQALNNPSMNEVYLLSTFKLQPKATVTLFGLYSTSDNSRFFEFTVMGRLNKASLRYLKSDGKLNSVIFSNIEIADGKQHTLLLHLSGLHRGSSSAELYLDCVPVDGVKDLPRPLLGASLNAGSVHLKTLQKRGQDSMDELKLVMGGTLSQISSIQECFMQQSEPGQQTGDVSRQLIGQITQMNQLLGELRDVMRQQVKETMFLRNTISECQAC

>XtTSP-5
MLSVALLSSFCIFFGSCQQLSGRGDVGPQLLTEMKETNSVLREVRELLKRQIEEITFLKNTVMECDAC

>GgTSP-1
MCAECRTQLPVGLPCLESCCPCSVYLVTPASLGKSRGDDDSVFDIFELTVRKGAGRRAPGVHLVKGPDTSSPAYRIEDASRIPAVSDSKFQDLLDIIHAEKGFILQATLRQAKKSRGTLLAVERKDGSGHVFSLVSNGKAGTLDLSLSGDGKQQLVSVEDALLATGHWKNITLFVQEDRAQLYVGCEKMENAELDIPIQNIFTRDLASSARLRIAKGGVNDNFQGLLQNVRFVFRTTLETIMRNKGCSSSTSAIITLDKPMNGSSPAIRTNYIGHKTKDIQAVCGFSCDELTNMFVELQGLRSMVTTLQDRVRKVTEENELIAKVVQITPGV

>GgTSP2
MLQRSRLLWLAVFITLWVSSDAQDDAKEEENTDLLQISNINRKTIGAKLFRGPDPAIPAYRFIRFDHIPPFKPEKLKKIVKLIRQNEGFILSATLRQRQSRGTILALEGPGISERQFEIISNGRANTLDLIYWVDGFQNVISLEDVDLADSQWKNLTVQVTGENYNLYVGCDLIDSFILEEPFYEQLKAENSRMYVAKGSIRENHFRGLLQNIHLIFDTSIEDVLRKKGCQRSQSTEVNTINESTEILHLSPAVTTEYVGEKTEKKAEFCDRSCEELGTMFTELTGLRIVVNNLADNLQKVSEENQIMWELIGPNKTL

>GgTSp-4
MQALINESPQQSHKIHGAIIDLLPYANKRVVTSFLQQALGDPTLNEVFLLSVFKLQPKSTTTIFDLYSPADDRQYFEFTVMGRMNKAFISAFIKAGMREKYQSHYRFPQNFAWSVIDYLSPSDSRSLYFVPVVLRYLKNDGRLNSVIFSNVHLADGKPHAVILWLSGLQQELCTIELYLDCLQVGAIQDLPKAFSTLLERSAAVELRTFLKKPEDTLDELKLVTGGTLAQARDLQDCFLQQIESAPQYTGDFNRQLMNQMVQMNQILGEVKDLLKQQVKETTFLRNTIAECQAC

>GGTSP-5
MISALAFVFLLCLSCPFSSCQQRRAGIEVGPEMLEEMRETNRVLMEVRDLLKQQIKEITFLKNTVMECDAC

>MmTSP1
MELLRGLGVLFLLHMCGSNRIPESGGDNGVFDIFELIGGARRGPGRRLVKGQDLSSPAFRIENANLIPAVPDDKFQDLLDAVWADKGFIFLASLRQMKKTRGTLLAVERKDNTGQIFSVVSNGKAGTLDLSLSLPGKQQVVSVEEALLATGQWKSITLFVQEDRAQLYIDCDKMESAELDVPIQSIFTRDLASVARLRVAKGDVNDNFQGVLQNVRFVFGTTPEDILRNKGCSSSTNVLLTLDNNVVNGSSPAIRTNYIGHKTKDLQAICGLSCDELSSMVLELKGLRTIVTTLQDSIRKVTEENRELVSEL

>MmTSP2
MLWALALLALGIGPRASAGDHVKDTSFDLFSISNINRKTIGAKQFRGPDPGVPAYRFVRFDYIPPVNTDDLNRIVKLARRKEGFFLTAQLKQDRKSRGTLLVLEGPGTSQRQFEIVSNGPGDTLDLNYWVEGNQHTNFLEDVGLADSQWKNVTVQVASDTYSLYVGCDLIDSVTLEEPFYEQLEVDRSRMYVAKGASRESHFRGLLQNVHLVFADSVEDILSKKSCQHSQGAEVNTISEHTETLHLSPHITTDLVVQGVEKAQEVCTHSCEELSNMMNELSGLHVMVNQLSKNERVSSDNQFLLELI

>MmTSP3
MEKPELWGVLALLLLCSYTCGSDLQVIDLLTVGESRQMVAVAEKIRTALLTAGDIYLLSTFRLPPKQGGVLFGLYSRQDNTRWLEASVVGKINKVLVRYQREDGKVHAVNLQQAGLADGRTHTALLRLRGPSRPSPGLQLYVDCKLGDQHAGLPALAPIPPAEVSGLEIRTGQKAYLRMQGFVESMKIILGGSMARVGALSECPFQGDDSIHNAVTSALQSILGEQTKALVTQLTLFNQILVELRDDIRDQVKEMSLIRNTIMECQVCG

>MmTSP4
MPAPRAAAAAFLLLHLVLQPWQRTSAQATPQVFDLLPSSSQRLNPSALQPVLTDPTLHEVYLISTFKLQSKSSATIFGLYSSSDNSKYFEFTVMGRLNKAILRYLKNDGKIHLVVFNNLQLADGRRHRVLLRLSNLQRGDGSVELYLDCAQADSVRNLPRAFSGLTQNPESIELRTFQRKPQDFLEELKLVVRGSLFQVASLQDCFLQQSEPLAATSTGDFNRQFLGQMTQLNQLLGEVKDLLRQQVKETSFLRNTIAECQAC


>MmTSP5
MGPTACVLVLALAILRATGQ GQIPLGGDLAPQMLRELQETNAALQDVRELLRHEVKEITFLKNTVMECDA C
 
>HsTSP1
MGLAWGLGVLFLMHVCGTNRIPESGGDNSVFDIFELTGAARKGSGRRLVKGPDPSSPAFRIEDANLIPPVPDDKFQDLVDAVRTEKGFLLLASLRQMKKTRGTLLALERKDHSGQVFSVVSNGKAGTLDLSLTVQGKQHVVSVEEALLATGQWKSITLFVQEDRAQLYIDCEKMENAELDVPIQSVFTRDLASIARLRIAKGGVNDNFQGVLQNVRFVFGTTPEDILRNKGCSSSTSVLLTLDNNVVNGSSPAIRTNYIGHKTKDLQAICGISCDELSSMVLELRGLRTIVTTLQDSIRKVTEENKELANEL

>HsTSP2
MVWRLVLLALWVWPSTQAGHQDKDTTFDLFSISNINRKTIGAKQFRGPDPGVPAYRFVRFDYIPPVNADDLSKITKIMRQKEGFFLTAQLKQDGKSRGTLLALEGPGLSQRQFEIVSNGPADTLDLTYWIDGTRHVVSLEDVGLADSQWKNVTVQVAGETYSLHVGCDLIGPVALDEPFYEHLQAEKSRMYVAKGSARESHFRGLLQNVHLVFENSVEDILSKKGCQQGQGAEINAISENTETLRLGPHVTTEYVGPSSERRPEVCERSCEELGNMVQELSGLHVLVNQLSENLKRVSNDNQFLWELI

>HsTSP3
METQELRGALALLLLCFFTSASQDLQVIDLLTVGESRQMVAVAEKIRTALLTAGDIYLLSTFRLPPKQGGVLFGLYSRQDNTRWLEASVVGKINKVLVRYQREDGKVHAVNLQQAGLADGRTHTVLLRLRGPSRPSPALHLYVDCKLGDQHAGLPALAPIPPAEVDGLEIRTGQKAYLRMQGFVESMKIILGGSMARVGALSECPFQGDESIHSAVTNALHSILGEQTKALVTQLTLFNQILVELRDDIRDQVKEMSLIRNTIMECQVC

>HsTSP4
MLAPRGAAVLLLHLVLQRWLAAGAQATPQVFDLLPSSSQRLNPGALLPVLTDPALNDLYVISTFKLQTKSSATIFGLYSSTDNSKYFEFTVMGRLSKAILRYLKNDGKVHLVVFNNLQLADGRRHRILLRLSNLQRGAGSLELYLDCIQVDSVHNLPRAFAGPSQKPETIELRTFQRKPQDFLEELKLVVRGSLFQVASLQDCFLQQSEPLAATGTGDFNRQFLGQMTQLNQLLGEVKDLLRQQVKETSFLRNTIAECQAC


>HsTSP5
MVPDTACVLLLTLAALGASGQGQSPLGSDLGPQMLRELQETNAALQDVRELLRQQVREITFLKNTVMECDAC
